# The evolution of the tree of life

**DOI:** 10.1098/rstb.2024.0091

**Published:** 2025-08-07

**Authors:** Molly Chen, Artem I. Kholodov, Laura A. Hug

**Affiliations:** ^1^ Department of Biology, University of Waterloo, Waterloo, Ontario N2L3G1, Canada; ^2^ Department of Data Science, University of Waterloo, Waterloo, Ontario N2L3G1, Canada

**Keywords:** tree of life, phylogenomics, taxon selection, evolution, diversity

## Abstract

The tree of life is one of the most important organizing principles in biology. Updates and revisions are historically derived from improved data capture, increasingly refined models of evolution and expanded taxon sampling. Tracing the changes in the tree of life over the molecular era (1990–present) highlights the evolution of biologists’ understanding of life on earth and serves as a foil placing the explosion of available data over this timeframe in context. Using current-day information, we explored the taxonomic growth captured in a tree of life through historic tree reconstruction. Data capture is now facilitating improvements in genome quality rather than expanding deep diversity, as the rate of novel phylum discovery is slowing for bacteria and archaea. Using dissimilarity metrics, the proportion of changes that each historic tree encompasses identified a diminishing influence of additional taxa on high-level topological revisions. No trees recapitulated current hypotheses for deep relationships on the tree of life, reflective of disadvantages associated with high taxon sampling and the divide-and-conquer methodologies required to analyse extremely large datasets. This work clarifies the effect of the interaction between data quality, data quantity and taxonomic diversity on our ability to construct a stable tree of life.

This article is part of the discussion meeting issue ‘Chance and purpose in the evolution of biospheres’.

## 1. Introduction

In 1857, Charles Darwin wrote to his friend Thomas Huxley, ‘The time will come I believe, though I shall not live to see it, when we shall have very fairly true genealogical trees of each great kingdom of nature’ (see https://www.darwinproject.ac.uk/ (accessed 28 October 2024)from [1]). A hundred years later, in 1957, Stanier, Doudoroff and Adelberg declared ‘...it is a waste of time to attempt a natural system of classification for bacteria’ [2], which seemed like a valid conclusion given the tools available at the time. The development of molecular methods in the late 1970s marked the beginning of a fresh approach in the on-going effort to resolve ‘fairly true genealogical trees’ for the diversity of life on Earth.

### (a) The timeline of the molecular tree of life: 1977–today

In 1977, Woese and Fox conducted the first proto-phylogenetic analysis exploring the full tree of life with molecular data [3]. Using 13 organisms’ 16S SSU rRNA, they generated radiograph fingerprints and determined association coefficients between members. This work clearly identified the archaea as a distinct, domain-level lineage, although Woese and Fox did not initially name this new group. In 1986, Pace, Olsen and Woese published their first three-domain tree and coined the term 'Archaebacteria' for the new prokaryotic division [4]. At the time, the prevailing model of life’s diversity was the five kingdoms hypothesis, with all prokaryotes collected under the kingdom Monera. This model was under scrutiny because it was clear that plants and animals had derived from single-celled ancestors and the degrees of difference between the five kingdoms were uneven, with the Monera representing substantially more divergence [5].

Very early on in molecular phylogenetics, two competing hypotheses emerged to challenge the existing dogma of the five kingdoms and a strong prokaryote/eukaryote divide: Woese’s three-domain tree, with Archaea as a distinct domain [4,5] and a two-domain tree, based on the eocyte hypothesis wherein the eukaryotes branched from within the Archaea, initially as a nearest neighbour to the Crenarcheaota [6,7]. Within the eocyte hypothesis, the position of the eukaryotes was revised as additional archaeal lineages were identified, with the extended eocyte hypothesis placing eukaryotes with the TACK superphylum (named both as in tacking a ship ‘in order to change course or direction’ and as an acronym of the four original phyla, the Thaumarchaeota (now Nitrososphaeria), Aigarchaeota, Crenarchaeota (now Thermoprotei) and Korarchaeota (now Korarchaeia)) [8,9]. The TACK superphylum is currently named the Thermoproteota under the Genome Taxonomy Database (GTDB) classification system [10].

In 2008, a third hypothesis was posited, where eukaryotes branched within the Archaea, but with a non-TACK, unknown closest relative. Yutin *et al.* predicted an intermediate archaeal lineage with eukaryotic features—an ‘uncharacterized archaeal lineage that acquired some euryarchaeal and crenarchaeal genes via early horizontal gene transfer’ [11]. This prediction encompassed features of two prominent eukaryogenesis hypotheses: endosymbiotic models, where the first eukaryote was an archaeal–bacterial chimera defined by the acquisition of the mitochondrion [12–14], and the archezoa hypothesis, where an amitochondriate proto-eukaryote already possessed eukaryotic cell features (nucleus, endomembrane system, cytoskeleton) prior to acquisition of the alpha-proteobacterial mitochondrion [15]. The discovery of the Asgardarchaeota in 2015 [16] echoed Yutin *et al*.’s hypothesis 7 years later [11] and unified these two theories—an archaeal host where characteristic aspects of eukaryotic cellular organization (e.g. cytoskeletal actin) had evolved prior to and independent of the mitochondrial symbiosis. The Asgardarchaeota place as nearest neighbours to the eukaryotes in many recent phylogenomic analyses [17–19], with some evidence for a branch point from the Heimdallarchaeia specifically [20].

### (b) Constructing universal trees of life—constraints and innovations

Changes to the structure of the tree of life have historically been a result of increased taxon sampling, improvement in gene(s) used and/or fit of the models used to quantify evolutionary change over billions of years [21]. Improvements to the accessibility and availability of sequencing data are major factors contributing to advances in phylogenomics. The NIH GenBank sequence database [22], which contained 606 sequences (totalling 680 338 bases) in its earliest recorded release in 1982, has expanded to over 250 000 000 sequences (3.68 × 10^12^ bases) as of August 2024 [23]. The RefSeq database, which represents a comprehensive, non-redundant set of annotated reference sequences, grew from just over 2000 to over 150 000 represented species in the past 20 years [24]. As sequencing depth in databases has increased and genomes have been resolved for more lineages, the marker gene sets available as ‘universal’, single-copy and conserved have increased [25,26]. More sophisticated models of evolution now take into account convergence over deep time, long branch attraction (LBA), heterogeneity across sites and differential rates of evolution within multi-gene analyses, leading to reduced artefacts and improved confidence measures [21], although resolution of deep divergences remains a challenge. A constraint on tree expansion remains computational complexity with large input matrices—as a result, trees are typically inferred with either many taxa or large concatenated alignments, but not both.

A bacteria–archaea phylogenomic tree containing 200 000 organisms and 387 marker genes was published in 2023 [27], using a filtered set of genomes from the GenBank and RefSeq genome databases. To construct such an enormous tree, Balaban *et al.* introduced uDance, a tree inference software using a ‘divide-and-conquer’ approach and a distributed computing method to infer a consensus tree from individual gene trees and partitioned sub-trees. This method allows for more efficient and rapid generation of trees compared with *de novo* tree construction from concatenated alignments.

Given modern sequencing technology and computational algorithms, it is now possible to process and integrate massive amounts of genomic sequencing data into phylogenomic analyses. However, it is not clear whether recent additions to genome databases are still remodelling our understanding of large-scale evolutionary relationships. It is possible that modern phylogenomics is close to achieving a consensus tree. If so, we would expect to see an asymptotic effect of increasing taxonomic sampling or genetic information towards changing the tree structure. To help answer this question, we reconstructed universal trees using current computational tools (uDance, FastTree [28]) and available genome assemblies at different timepoints over the past 25 years. We divided GenBank genome data into 5-year intervals from 1999 to 2024 and generated a tree from the available sequences at the time using modern methodology. In doing so, we offer a retrospective on how our perspective on the tree of life has evolved over the molecular era.

## 2. Methods

### (a) Analysis of published trees

We conducted a thorough literature review of published universal trees of life, starting from Pace *et al.* in 1986 [4], which presented the first tree of life using molecular data. From each article, we compiled the available data on the number of organisms included on the tree, the genes used, the molecule type (nt or aa) and the final length of the alignment in columns. From this, we calculated the total information in the tree, as nucleotides (e.g. # columns multiplied by # taxa, and then multiplied by 3 for amino acid alignments). We also noted the reported topology and any key innovations in model or inference method (see electronic supplementary material, table S1 for a full summary).

Articles were identified through keyword search, from published reviews of the history of the tree of life (e.g. [1,29]) and from the references within articles already included in the analysis. Articles were included in this meta-analysis if they presented trees containing representatives from the three domains (Bacteria, Archaea, eukaryotes) and either the data contained in the tree(s) were reported in the article, or the alignments used to infer the tree(s) were publicly available. When multiple universal trees were included in a publication, either the largest dataset or the tree upon which conclusions were based (in cases where models were being tested) was selected for inclusion. If two trees were discussed equally, both were included. Geneious was used to view alignments when required [30]. For cases where data were not available, corresponding authors were approached to request the information.

A secondary set of notable phylogenomic trees from published papers was included in this review. These trees represent large-scale explorations of single- or dual-domain-level divergence and provide context for the scale of data processed at equivalent points in time to three domain datasets (see electronic supplementary material, table S1).

### (b) Data collection

The genome dataset used for reconstructing historical trees was curated from the GenBank sequence database [22]. Genomes were grouped into five sets based on the release date (inclusive): 1999−2004, 1999−2009, 1999−2014, 1999−2019 and 1999−2024. To minimize redundancy, the following criteria were independently applied to all sets to filter genomes down to one unique representative per genus, based on NCBI taxonomy. (i) The most recent reference genome was kept if available. (ii) If there was no reference genome, the most recent full/complete genome was kept, otherwise the genome with the highest CheckM completeness was kept [31]. (iii) Genomes without a classified genus were assumed to be unique. Additional filtering to remove viral genomes, metagenome assemblies and synthetic constructs from the initial database was also performed based on GenBank metadata associated with each entry (see electronic supplementary material, table S2 for intermediate and final numbers of genomes available at each timepoint).

Sequences were downloaded with the NCBI datasets tool in August 2024. Out of 2 199 464 GenBank entries under ‘genome’, 124–333 161 were selected for tree construction depending on the date window selected (electronic supplementary material, table S2). Data filtering, sorting and aggregation were done in Python (v.3.8.8) with the pandas library [32]. All accessions for genomes included in trees, including their taxonomy assignments, are included in electronic supplementary material S1, available in excel format on the Open Science Framework under DOI (10.17605/OSF.IO/NH4VF).

### (c) Tree inference

Only GenBank assemblies with available protein (.faa) files were used for tree construction (electronic supplementary material, table S2). In cases where there was no GenBank protein file, we performed a separate search using the equivalent RefSeq accession and substituted with the RefSeq protein file if it was available.

HMMER v.3.2.1 [33] was used to identify marker genes, based on the 16 ribosomal genes used in the [34] tree of life, selected to be comparable with that prior high taxonomic-sampling inferred tree [34]. Hidden Markov model (HMM) profiles were retrieved from the protein families (Pfam) database [35] and used as search seeds in HMMER to identify best-scoring hits from each genome for each of the 16 ribosomal protein genes. To minimize false positives from mitochondrial and chloroplast DNA in eukaryotes, we constructed a separate HMM profile for the eukaryotic-specific version of each gene. Eukaryotic genomes were processed separately and resulting hits were merged with the bacterial and archaeal datasets. In cases where HMMER identified multiple hits for a marker gene within a single organism (which is common in eukaryotic genomes), the lowest e-value hit was kept for alignment.

Multiple sequence alignments (MSAs) were generated with MUSCLE v.5.2 [36] for all marker genes, using the Super5 algorithm to speed up alignment of large datasets. MSAs were stripped of positions containing >90% gaps in Geneious [30] and manually visualized to filter out false positives based on sequence divergence and long branches on single gene trees. Tree construction with quality filtered MSAs was done with uDance [27], with a minimum occupancy threshold of 8 genes (50%) and tree inference method set to RAxML-NG [37]. A backbone of 22−1000 taxa (based on dataset size) was independently inferred for all trees from the input genomes. Target backbone size was set to 16.5%–20% of input sequences up to a maximum of 1000, with backbone taxa selected by uDance.

The performance of the uDance divide-and-conquer method was compared to de novo tree construction using FastTree [28], which uses neighbour joining in combination with approximate Maximum Likelihood methods to infer trees, with nearest-neighbour interchanges and subtree pruning-regrafting to explore alternative topologies. We concatenated the trimmed alignments of the 16 ribosomal marker genes for all genome datasets used in the uDance analyses and inferred trees using FastTreeMP (v.2.1.11) under default settings.

Trees were visualized using the online Interactive Tree of Life (iTOL) [38]. For large tree files (>10 000 organisms), Dendroscope [39] and Biopython [40] were used to process and format labels. Labels and annotations were assigned based on taxonomy data from the GTDB [41]. For eukaryotes, NCBI taxonomy data from the GenBank FTP website [10] were used instead of GTDB.

All trimmed alignments and final uDance and FastTree trees are available on the Open Science Framework [42].

### (d) Tree comparisons

Dissimilarity metrics between reconstructed trees were evaluated with Visual TreeCMP [43] and the Environment for Tree Exploration (ETE) (v.3.1.3) library in Python [44]. Trees were pruned to the 71 taxa (genus level) shared between all five trees. The Robinson–Foulds (RF) (both weighted and unweighted), Matching Triplet, and Matching Split distances were calculated between chronologically adjacent trees using the 71 overlapping taxa. Additionally, the unweighted Robinson–Foulds distance was calculated using all overlapping taxa (105–11 533 in total) between adjacent pairs of trees.

## 3. Results

### (a) Published trees through time

**Figure 1.**
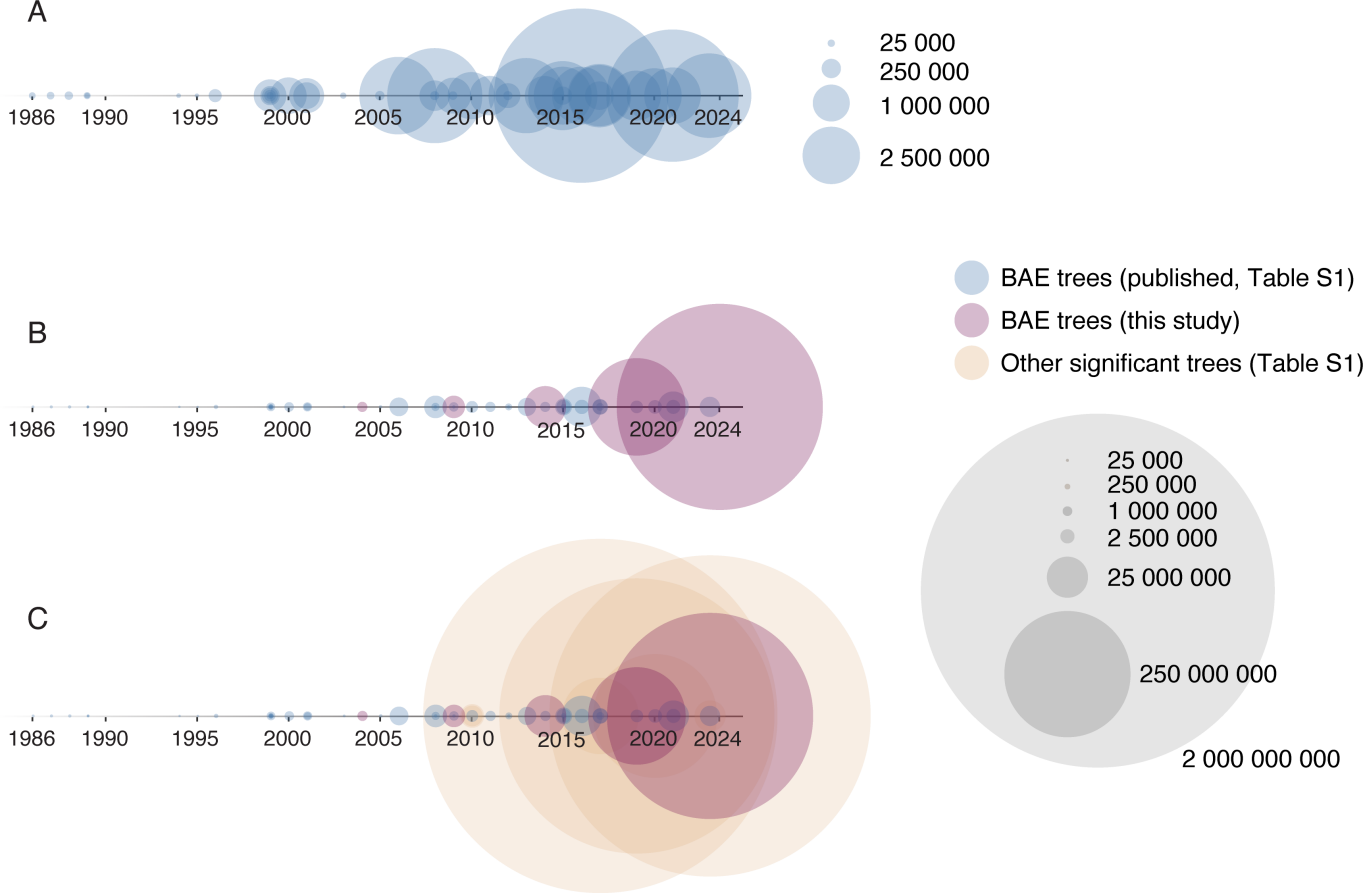
Timeline of universal trees of life, by total information included in the trees**.** Circles represent individual universal trees of life, with information calculated as # taxa × # alignment positions (×3 for amino acid alignments). (A) Published universal trees of life. (B): Published universal trees of life and reconstructed trees of life from this study (new scale). (C): Timeline in B, with other, large-scale two-domain trees included (orange), same scale as B.

**Figure 2.**
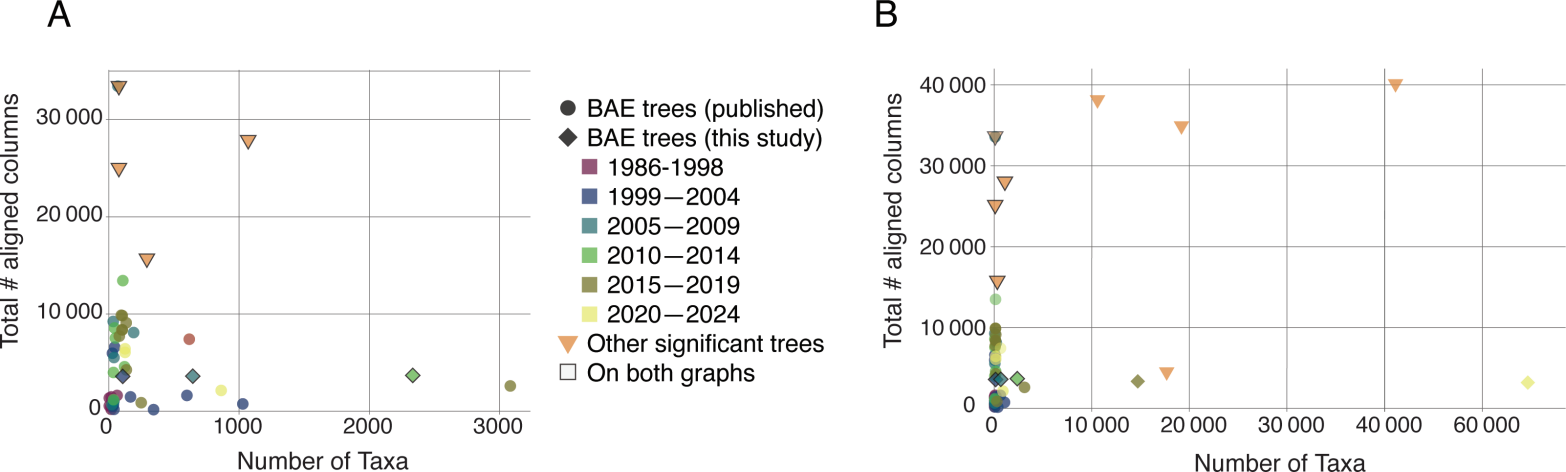
Taxon sampling versus molecular information used to construct universal trees of life. (A) All published universal trees and subsets of reconstructed trees from this study and from other significant two-domain trees. (B): Expanded view, including all reconstructed trees from this study (diamonds) and the full set of other significant two-domain trees included in figure 1 and electronic supplementary material, table S1. All universal trees are coloured by the 5-year window within which they were generated. Reconstructed (diamond) and two-domain (triangle) trees included on both plots are outlined to facilitate connection between the two *x*-axis scales.

Initial trees exploring the relationships between the Bacteria, Archaea and eukaryotes were marked by low taxon sampling (4–60 taxa) and a limited number of genes. The 16S SSU rRNA gene dominated early trees [4,45,46], with others using elongation factor EF-Tu subunits or ATPases ([47–49], see electronic supplementary material, table S1 for curated data from published trees of life). The amount of data used to infer a tree increased markedly between 1999 and 2001, with a shift to multiple protein markers combined with modest taxon sampling (figure 1A–C, Katoh *et al.*: 39 marker genes and 28 taxa [26], Brown *et al.*: 23 marker genes and 45 taxa [25]). From there, improvements in tree inference datasets derived from either increased taxon sampling or refined marker gene selection, but rarely both (electronic supplementary material, table S1, figure 2A).

Identifying additional universal, vertically inherited, single-copy marker genes has proven difficult, meaning taxon sampling is currently the more amenable avenue to increasing the information included in a tree inference. There has been a slow increase in the number of taxa included on a universal tree, progressing from 4 to 64 taxa in trees between 1986 to 1999, to more recent trees depicting 618 and 863 taxa [18,50] (figure 2A, electronic supplementary material, table S1). The tree from Hug *et al.* [34], published nine years ago in 2016, is an outlier to this trend, with 3083 taxa—the highest taxon sampling identified from our literature review.

### (b) Reconstructed trees

Given that taxon sampling is the more tractable mechanism to add data to a tree inference, and with the development of the uDance software to handle extremely large phylogenomic datasets, we decided to construct historical trees using genome sequencing information available during each time interval. Datasets of GenBank genomes were generated in five-year incremental windows: 1999−2004, 1999−2009, 1999−2014, 1999−2019 and 1999−2024. We selected a set of 16 ribosomal proteins (16 RP) as marker genes and generated alignments for each incremental dataset. The 1999−2004 and 1999−2014 dataset sizes are in-line with those underlying published trees in those time windows (figure 1B), but with the rapid explosion of sequence information from 2014 onwards (electronic supplementary material, figure S1), the later datasets contain substantially more data than their contemporary trees (figure 1B). Our reconstructed trees contain less information than large single- or dual-domain trees in those time windows (figure 1C), which is a function of the smaller marker gene set used in our analyses (figure 2B).

**Figure 3.**
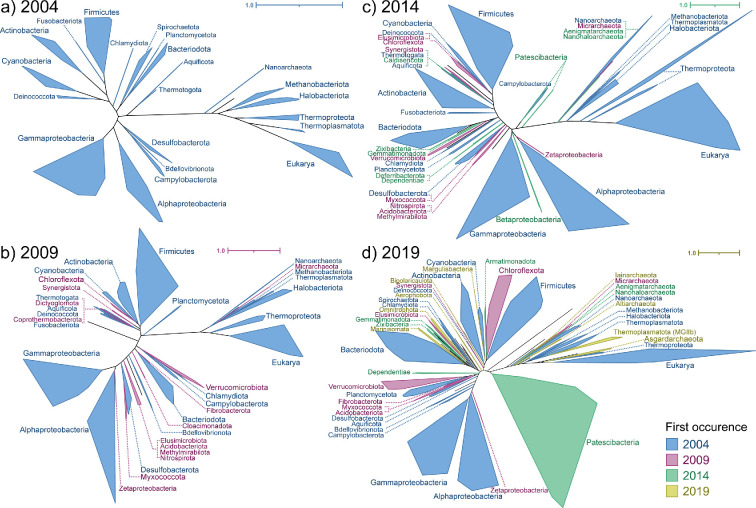
Reconstructed trees based on historical genomic sequencing data from 1999 to 2019. Trees are shown chronologically, each capturing an additional 5 years of data from the GenBank genome database. Clades are annotated at the phylum level with GTDB nomenclature, except for eukaryotes as they do not have associated GTDB representatives. To facilitate legibility, not all available phyla in trees b–d are shown. Colours indicate the reconstructed tree in which the phylum first appeared, in chronological order.

**Figure 4.**
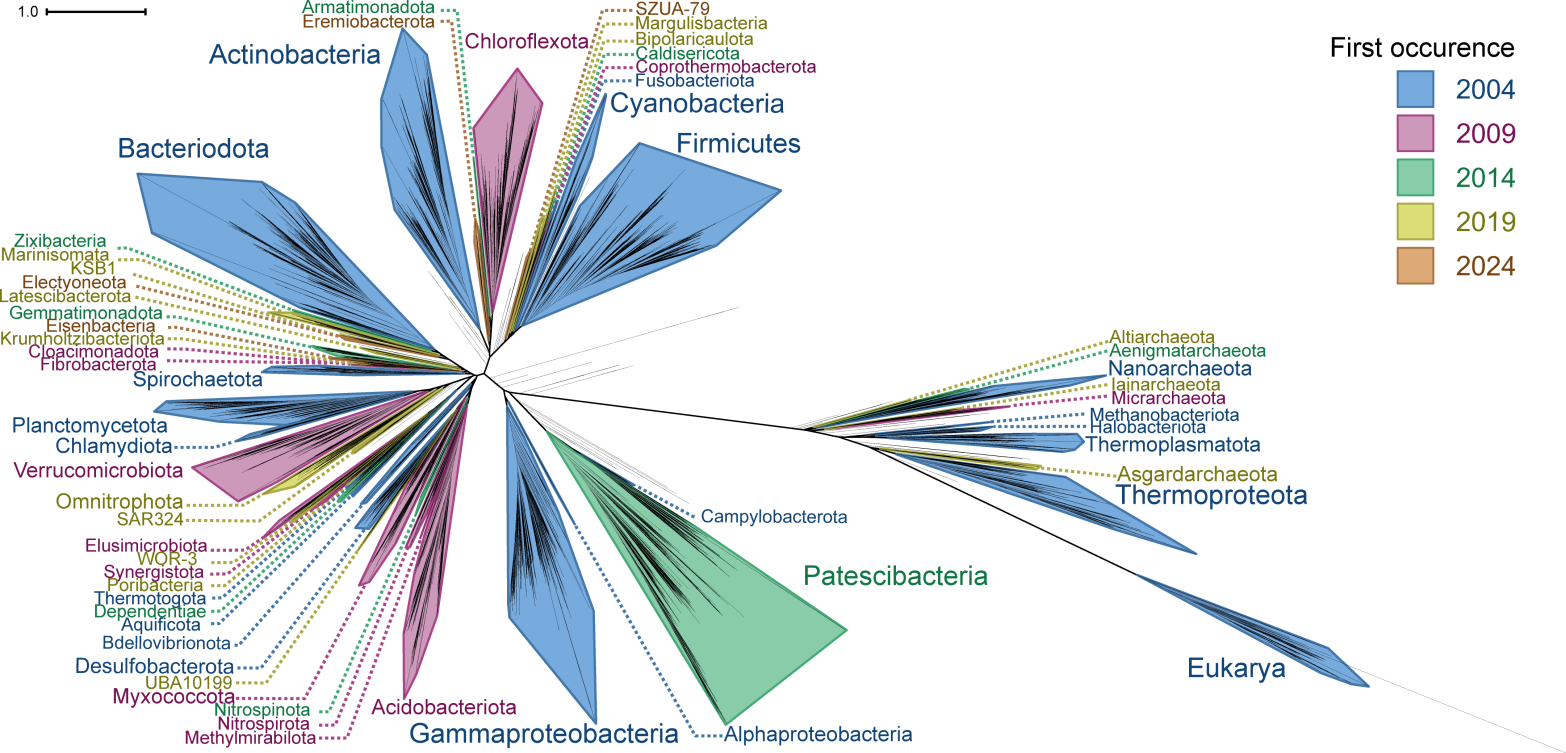
Reconstructed universal tree of life using genome sequencing data from 1999 to 2024**.** The tree was constructed using available sequences in the GenBank sequence database as of August 2024. A total of 69 496 organisms were placed by uDance. Annotations are based on phylum-level taxonomy assigned by GTDB. Phyla with 35 or more representatives are shown, as well as selected phyla present in figure 3. Colours indicate the reconstructed tree that the phylum first appeared in, in chronological order.

Five historically reconstructed trees were inferred from the 16 RP datasets using uDance (referred to as the 2004, 2009, 2014, 2019 and 2024 trees for the end date of each dataset, respectively) (figures 3 and 4). These trees included 107–69 496 taxa (table 1), with the first instance of each named phylum highlighted with its associated time window. FastTree was used to generate comparison trees from concatenated alignments (electronic supplementary material, figures S23). We initially annotated trees based on NCBI-assigned phyla but found that clades in reconstructed trees, even for the 2004 tree, agreed more robustly with the GTDB taxonomy. Therefore, we mapped all placed organisms to their respective GTDB entry if one was available and annotated trees with GTDB phylum level groupings. In the case of eukaryotes, we used NCBI taxonomy assignments for the purpose of counting the number of unique phyla and genera represented in reconstructed trees (table 1).

**Table 1.** Distribution of organisms in historic reconstructed trees. Counts are based on the total organisms successfully placed on trees in figures 3 and 4. Unique phyla, unique genera and classified organisms (phylum level) are based on taxonomy assignments within the Genome Taxonomy Database (GTDB) [41,51], with the exception of the Eukarya, which do not have equivalent GTDB classifications and were categorized based on NCBI taxonomy.

Release date range (inclusive)	Archaea	Bacteria	Eukarya	Unique phyla	Unique genera	Classified organisms	Total organisms	Total information (as nt)
1999−2004	15	85	6	25	106	106	107	1 149 501
1999−2009	45	525	74	55	614	638	645	6 969 870
1999−2014	146	1795	380	77	2109	2299	2334	25 774 362
1999−2019	1119	12 730	548	138	5962	11 664	14 706	147 883 536
1999−2024	6823	58 826	1781	202	14 194	39 743	69 496	673 833 216

The reconstructed trees showed erratic topologies and unexpectedly high impacts of LBA and other artefacts. In addition, we found that stochasticity in the uDance algorithm would often produce very different output trees given the same initial inputs. There was substantial variability in tree topologies at the phylum and domain levels, including the branching point of the Archaea for different trees inferred from the same dataset. Additionally, a large clade would often be left out of the final tree entirely, with all genomes belonging to that group marked as ‘unable to be placed’. Examples include the Spirochaetota in 2014 (figure 3C) and two separate inferences of the 2014 tree where the Cyanobacteria and Actinobacteria were left out, respectively. All fungi were missing from the final 2019 tree (figure 3D), as were most Alphaproteobacteria from the 2024 tree (figure 4). The trees showed shared topologies only at the highest level: each of our historically reconstructed trees supports a two-domain hypothesis. Even in the 2004 reconstructed tree (figure 3), with 107 organisms and only 15 archaea and 6 eukaryotes, the Archaea are not a monophyletic group distinct from eukaryotes. From this, we proceeded with a comparison of timelines of discovery of microbial lineages rather than an exploration of tree topologies.

The historical trees provide a fascinating foil for examining changes in the rate of discovery of microbial lineages. Despite the massive increase in the number of genomes available for tree reconstruction over time, the amount of represented diversity does not scale proportionally. The number of unique phyla more than doubled from the 2004 to the 2009 tree (table 1, figure 3), but the vast majority of organisms (90.4%) in the 2009 tree were placed into existing phyla. Early trees (2004−2014) also saw a proportionally higher increase in the number of eukaryotes compared with bacteria and archaea (table 1), a trend that was then reversed with the advent of environmental sequencing , which predominantly recovered bacterial and archaeal genomes. From a historical standpoint, the 2014−2019 transition is the point when populating databases with genome sequences from known/cultured organisms had reached a steady rate. A new wave of rapid growth in diversity occurred in this transition, focused on the discovery of new species and phyla from environmental sequencing and metagenomic data. For example, some of the change between the 2014 and 2019 trees was driven by the 1659 new placements added from the Patescibacteria (Candidate Phyla Radiation) (figure 3C-D). A minor proportion of the observed increases in taxonomic diversity stem from reclassifications of existing diversity: in this analysis, the GTDB nomenclature was applied to all bacterial and archaeal genomes regardless of their date of deposition to GenBank. Within this nomenclature shift, there have been reclassifications that increase taxonomic diversity without changing the genomic diversity of the database, including cases like the division of the genus *Lactobacillus* into 25 genera (23 novel) without changing the genomic information in GenBank [52].

Analysis of dissimilarity metrics between adjacent trees does not show a clear trend. Only weighted Robinson–Foulds distance, which takes branch length into account, shows a slight increase in dissimilarity in trees over time (table 2). Unweighted metrics such as RF and Matching Split remain relatively similar across adjacent trees (table 2). We were restricted to using only the shared overlapping taxa between all five reconstructed trees because Visual TreeCMP cannot calculate normalized results past trees of size 1000. However, we did calculate the normalized RF distances using pairwise overlapping taxa of adjacent trees in the Python ETE (v.3.1.3) toolkit [44], and observed similar results (table 2). Under RF, Matching Split and both normalized RF calculations, the 2014−2019 tree comparison shows the highest dissimilarity. For RF Weighted and MatchingTriplet, the 2019−2024 comparison had the highest values.

**Table 2.** Dissimilarity of common taxa between historically reconstructed trees. Pairwise comparisons were made between chronologically adjacent trees. For columns 3−7 (‘Overlapping taxa...’), trees were pruned to the 71 (genus-level) taxa that are shared between all five trees. For columns 8 and 9 (‘Pairwise overlapping taxa’), trees were pruned to the overlapping taxa that are shared between each successive pair. Only the normalized RF value rather than the Weighted value is reported as the number of common taxa differs in each comparison.

Comparison		Overlapping taxa between all trees (71 total)					Pairwise overlapping taxa	
tree1	tree2	RFWeighted (0.5)	RF (0.5)	MatchingTriplet	MatchingSplit	Normalized RF	Common taxa (genus level)	Normalized RF
2004	2009	6.60	25	17 039	140	0.37	105	0.29
2009	2014	7.83	22	15 665	154	0.33	612	0.31
2014	2019	9.35	35	15 294	153	0.52	1850	0.43
2019	2024	13.83	32	18 447	140	0.47	11 533	0.35

## 4. Discussion

### (a) Published trees through time

The amount of information included in universal trees has not kept pace with increases in sequence availability or taxonomic expansion. From 2008 onwards, the amount of information used to infer published universal trees has been static, with two exceptions (figure 1A, one case with higher marker gene numbers [11] and one case with higher taxon sampling [34]). In the meantime, substantially larger datasets have been used to generate phylogenomic trees for one or two domains, including Archaea–Bacteria and Archaea–eukaryote pairings (figure 1C, [20,51,53–56]). Total marker genes included in a universal tree have stabilized in the ~35–45 gene range, with the notable exception of Yutin *et al*. in 2008 including information from 355 genes [11], a feat leveraging *post-hoc* consensus of individual gene trees to reduce computational complexity (figure 2A). There are robust marker gene sets with over 100 genes for the Archaea and the Bacteria [57] that have led directly to new taxonomies for these Domains through the GTDB, containing substantially more information than any published universal tree (figure 2B). GTDB does not currently include marker genes or a revised taxonomy for the eukaryotes, and the bacterial and archaeal marker gene sets have imperfect overlap. The GTDB has thus enabled many phylogenomic analyses, but not those encompassing a universal tree of life.

### (b) Reconstructed trees

Our reconstructed trees encompass the available taxonomic diversity at a given timepoint, with a stable set of protein marker genes (16 RP). Originally, we intended to examine the impact of increased taxon sampling (and a related reduction in overall branch lengths) on universal tree inference. The reconstructed trees, however, did not provide sufficient support values nor the consistency to allow this level of discussion beyond the highest taxonomic levels. For instance, the observed stable two-domain topology suggests that database limitations on taxon sampling would not have been a determining factor for tree topology discrepancies in the 2004−2014 window (for trees with broad taxonomic sampling). Instead, marker gene selection and methods for tree construction are likely to have more strongly impacted tree inferences . SSU rRNA genes have shown mixed domain topologies in the past [7,9,34], with more frequent three-domain tree topologies resolved—even in trees with very high numbers of taxa [34]—compared with protein data, which has more frequently supported a two-domain topology (electronic supplementary material, table S1).

In contrast, the placement of the Patescibacteria indicates issues with phylum-level placements from the uDance analyses. In our reconstructed trees, the Patescibacteria (or Candidate Phylum Radiation) are identified as the sister taxon to rest of the Bacteria in all reconstructed trees that contain them (2014−2024, figure 3C-D, figure 4), in agreement with some previously published trees [34,58]. Placement of Patescibacteria on universal trees is a contentious topic. Recent studies have updated bacterial nomenclature to reflect two major lineages based on the evolution of the cell envelope: the Bacillati (formerly Terrabacteria) and Pseudomonadati (formerly Gracilicutes) [59,60]. Under this model, the Patescibacteria are placed within the Bacillati (single membrane, monoderm) kingdom next to the Chloroflexota [60–62]. Within this model, published trees consistently place the kingdom Bacillati (Firmicutes, Actinobacteria, Cyanobacteria, Patescibacteria and others) as basal in the bacterial tree compared to the Proteobacteria [19,27,34,50,63–65]. The basal placement of Patescibacteria as isolated from the other Bacillati lineages in universal trees is likely owing to LBA [60–62], which is a common artefact of universal trees that remains a challenge to resolve. One recently published bacteria–archaea tree showed the updated placement of Patescibacteria within the Bacillati [66]. This study also found that differences in taxonomic representation of groups can bias and can dramatically impact trees. The smaller sample size (1650 curated genomes) and balanced taxon sampling at the family level used in this study suggest that data quality is far more important than quantity (of genomes) for inferring universal trees. However, smaller sample sizes and taxon balancing may introduce their own biases; many genomes within the Patescibacteria that were not classified below the phylum level were excluded in this analysis [66], which may have obscured additional diversity within this clade that was not represented in the final tree.

Most inconsistencies between our high-level tree topologies, both between our historic trees and those in the literature, were owing to the fluctuating placement of undersampled phyla and their associated long branch lengths. These findings were likely influenced by the previously discussed effects of uneven taxon sampling [66], among other methodological concerns discussed below. When making universal trees, accurate placement of higher-order clades may require a minimum number of representatives for each group of interest to justify inclusion in the final tree, or, better, balanced representation at a desired taxonomic level as demonstrated by Martinez-Gutierrez and Aylward [66]. In contrast, clades with many sequenced representatives (e.g. Gammaproteobacteria, Firmicutes, Patescibacteria in the 2019 and 2024 trees) could be pruned to a smaller set of organisms when constructing universal trees, while still maintaining enough information to place these groups accurately.

Dissimilarity metrics (table 2) were relatively stable between chronologically adjacent pairs of reconstructed trees. The 2014−2019 trees have the highest RF dissimilarity. Growth in the number of unique phyla available in genome databases peaked during this time period (highest in 2016; electronic supplementary material, figure S1), then began to slow through to 2024. When the difference in initial tree size is considered, there was a clear diminishing impact from adding additional data to the tree. For instance, the Matching Split value (in terms of the shared 71 genera) is identical between 2004−2009 (an increase of 538 organisms) and 2019−2024 (an increase of 54 790 organisms). Furthermore, 99.3% of classified organisms in the 2024 tree were placed into existing phyla (present in 2019), which is an even higher proportion than the 90.4% in the 2004−2009 tree comparison. Peak growth in newly described genera occurred in 2020 (electronic supplementary material, figure S1) and has also slowed down in recent years. It is important to note that many sequenced genomes in GenBank do not yet have a genus (including placeholder names) as recognized by the GTDB, so observed growth in this metric may be partly owing to assigning names to existing database entries rather than the discovery of new organisms.

### (c) uDance for larger-scale tree inference

uDance is an appealing software for inferring larger-scale universal trees because it reduces computational requirements substantially compared with other tree inference programmes. When compared with FastTree—one of the only *de novo* methods that is scalable to the amount of sequencing information used in this study—we found that the two algorithms performed similarly with smaller datasets (2004, 2009, 2014), but that the uDance results were stronger with large datasets (2019, 2024; electronic supplementary material, figures S23). FastTree reconstructed trees become increasingly erratic as more genomes are included, with an increase in long, poorly supported branches and inaccurate placements. Notable examples in the 2024 tree include the Alphaproteobacteria being paraphyletic and the placement of many DPANN archaea within the Patescibacteria (electronic supplementary material, figure S3).

The difference in relative tree quality is likely because FastTree placed many more organisms than uDance, especially in the 2019 and 2024 datasets (electronic supplementary material, table S2). It was not possible to place over 40% of genomes in the final 2024 uDance tree, while only 10% were excluded from the 2024 FastTree tree. The rapid increase in available genomes from 2014 onwards is marked by an average reduction in genome quality as the proportion of deposited genomes that are closed and complete decreased. The tree partitioning method of uDance is more effective at filtering poor-quality (e.g. incomplete, contaminated) genomes than manual inspection of concatenated alignments. uDance has multiple checkpoints where genomes can be removed owing to low confidence, as it filters based on individual gene trees as well as partitioned organism trees. Unplaceable organisms are also determined by incompatibility of nodes between partition subtrees as they are stitched, providing another level of quality control. It also incorporates algorithms such as TreeShrink [67] to remove long branches as subtrees are combined. These factors appear to improve the signal-to-noise ratio massively in large, relatively uncurated datasets.

However, there are several inherent issues with the uDance approach that need to be addressed by researchers. The uDance method of inserting partition trees onto a backbone exacerbates the effect of undersampling within a dataset. The source of these issues is tied to the selection of the initial backbone. Although uDance allows for changes to the backbone as query sequences are added, from our tests, it appears that the final tree is still heavily biased towards the structure of the initial backbone. Reconstructed trees behave more like trees of size 22−1000 (the size of their backbone) rather than the size of the final tree. Backbone selection bias may contribute to the observed dissimilarities between reconstructed trees, as well as discrepancies between reconstructed and published trees. Given that FastTree inferred 2004−2014 trees that were comparable with uDance, it may be preferable to infer trees using concatenated alignments on smaller (<25 000 000 total nucleotide information) datasets to avoid backbone sampling bias. For larger datasets that benefit from the uDance divide-and-conquer method, it is critical that the initial backbone accurately captures the desired scope of diversity within the dataset. Ideally, the user selects a set of backbone organisms with appropriate diversity and adequate sampling depth. Other inconsistencies in reconstructed trees that are not directly tied to but probably exacerbated by the uDance algorithm arise from the limited marker gene set and common artefacts of universal phylogenomic trees themselves (e.g. LBA).

Results from both reconstructed and published trees suggest that applying a one-size-fits-all approach to constructing large scale, accurate universal trees may not be possible. However, tools such as uDance could, in combination with improved data curation, balanced taxon selection and backbone selection, be able to leverage the full potential of using divide-and-conquer algorithms to construct accurate, large-scale universal trees.

## 5. Conclusions

Universal trees of life have not been keeping pace with the expansion of available taxonomic data and remain limited by universal marker genes appropriate for modelling deep evolutionary events. At the same time, current additions to genome databases are not expected to affect major phylum/domain level diversity. The majority of new classified genomes (99.3% in the past 5 years) are additional representatives from existing groups. Only the discovery of some untapped source of diversity (novel environments, lineages below current detection limits) is expected to alter a trend of increased sampling depth having diminishing returns on higher-order taxonomic diversity.

Reconstructed trees leveraging public genome sequences represent historical snapshots of the breadth of diversity known to humanity over the molecular era and they reflect advancements in terms of our understanding of life on earth. However, properly resolving phylogenetic relationships between major groups cannot be achieved solely by adding additional genomes to universal trees. From our analyses and matching conclusions from the field, when it comes to tree construction, data quality and methodology matter more than quantity of taxa. The following improvements would generate more robust and reliable trees with less data than used here, but still more information than recently published trees: more stringent quality filtering metrics for genome selection, appropriately sampling diversity to include reasonable representation of all groups, and—specific to uDance-based analyses—control over the initial backbone taxon selection to ensure that all groups are included. We predict that future advancements will focus on developing and refining methods of data analysis, which will continue to change our understanding of the structure of the universal tree of life.

## Data Availability

Supplementary Data File 1 containing all genome information for the trees, as well as all tree files and alignment files used, are available on the Open Science Framework [42]. Supplementary material is available online [68].
